# Isolated Lymphomatoid Granulomatosis of the Central Nervous System Mimicking Trigeminal Neuropathy, Bell’s Palsy, and Glioblastoma in an Epstein-Barr-Negative Immunocompetent Host: A Case Report

**DOI:** 10.7759/cureus.45309

**Published:** 2023-09-15

**Authors:** Yohannes Ghenbot, John Arena, Susanna Howard, Connor Wathen, Mert Marcel Dagli, Patricia Zadnik, Ilya M Nasrallah, Ernest Nelson, Amy Pruitt, Eric Zager

**Affiliations:** 1 Department of Neurosurgery, University of Pennsylvania Perelman School of Medicine, Philadelphia, USA; 2 Department of Radiology, University of Pennsylvania Perelman School of Medicine, Philadelphia, USA; 3 Department of Pathology, University of Pennsylvania Perelman School of Medicine, Philadelphia, USA; 4 Department of Neurology, University of Pennsylvania Perelman School of Medicine, Philadelphia, USA

**Keywords:** isolated lymphomatoid granulomatosis, cranial neuropathy, epstein-barr virus, cns lymphoma, cns lymphomatoid granulomatosis

## Abstract

Lymphomatoid granulomatosis is an Epstein-Barr virus-associated lymphoproliferative B-cell neoplasm that typically involves multiple organ systems. This disease is exceedingly rare when confined to the central nervous system (CNS), usually presenting as a mass lesion or diffuse disease, with no existing standard of care. We present the case of a 67-year-old patient who had a unique and insidious course of isolated CNS lymphomatoid granulomatosis. The disease first presented with cranial neuropathies involving the trigeminal and facial nerves that were responsive to steroids both clinically and radiographically. Two years later, the disease manifested as a parietal mass mimicking high-grade glioma that caused homonymous hemianopsia. The patient underwent craniotomy for resection and was treated with rituximab after surgery. The patient has achieved progression-free survival more than three years after the surgery. Surgical debulking and post-procedural rituximab resulted in favorable survival in a case of isolated CNS lymphomatoid granulomatosis. An intracranial mass preceded by steroid-responsive cranial neuropathies should raise suspicion for lymphoproliferative disorder.

## Introduction

Lymphomatoid granulomatosis is a mature, extranodal, lymphoproliferative, B-cell neoplasm associated with Epstein-Barr virus (EBV) [1]. On histology, it is both angiocentric and angiodestructive, with a polymorphic infiltrate in which T lymphocytes predominate [2]. Disease pathogenesis is hypothesized to involve impaired surveillance of EBV-infected B-cells, as patients with lymphomatoid granulomatosis have reduced quality or quantity of cytotoxic CD8+ T cells [3]. This multisystem disease most frequently involves the lungs. It is rare for disease to be confined to the central nervous system (CNS). Recent review articles have found fewer than 50 reported cases [4].

Isolated CNS lymphomatoid granulomatosis has no pathognomonic clinical, radiographic, or laboratory characteristics, requiring histology for diagnosis [5]. Radiographically, it is difficult to distinguish lymphomatoid granulomatosis from other mass lesions of the brain despite advanced magnetic resonance imaging (MRI) and positron emission tomography (PET) scan, and preliminary diagnoses often include high-grade glioma, cerebral metastases, and inflammatory or infectious processes. Lesions are typically multifocal and cortically based. They may also have a diffuse appearance or involve subcortical white matter, basal ganglia, thalamus, sella, cerebellum, spinal cord, or cranial nerves, commonly causing cognitive decline, headache, and epilepsy [4].

Here, we present a unique case of lymphomatoid granulomatosis. Our patient had an atypical, insidious course with repeated neuroimaging, first presenting with cranial nerve involvement, and later presenting with a mass lesion.

## Case presentation

**Figure 1:**
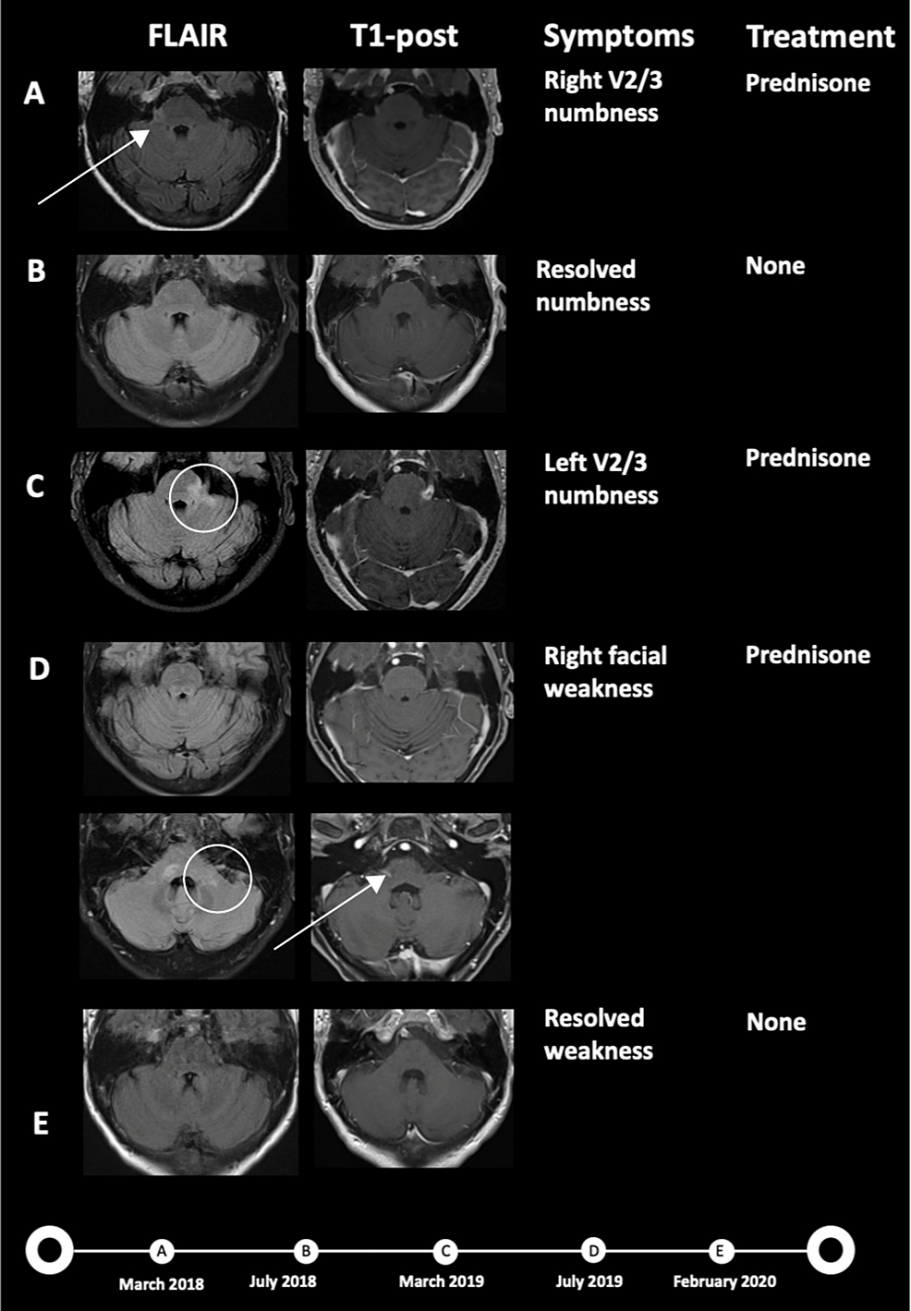
Clinical presentation: Imaging, symptoms, treatment, and timeline. The patient initially presented with right V2/3 sensory changes and was found to have fluid-attenuated inversion recovery (FLAIR) signal abnormality and enhancement at the trigeminal root entry zone (Arrow A). Symptoms improved after prednisone monotherapy and follow-up imaging showed resolution of FLAIR signal abnormality and enhancement (B). She later experienced new left-sided V2/3 sensory changes and was found to have an abnormal FLAIR signal and enhancement of the left trigeminal root entry zone (Circle C), treated with prednisone (C). She later developed a right facial palsy, treated with steroids. MRI revealed FLAIR signal abnormality and abnormal enhancement (Arrow D) involving the cisternal, canalicular, and labyrinthine segments of the right facial nerve. Previous FLAIR signal abnormality in the left trigeminal root entry zone (Circle D) resolved (D). Her facial weakness and FLAIR signal abnormality resolved on follow-up imaging (E). There was no abnormal signal in the right parietal lobe from March 2018 to February 2020 (not shown).

A 60-year-old female presented in March 2018 with right facial numbness in the maxillary nerve (V2) and mandibular nerve (V3) distributions. MRI identified an abnormal T2-weighted fluid-attenuated inversion recovery (T2 FLAIR) signal and enhancement in the right trigeminal root entry zone (Figure 1, Panel A). She was treated for one week with prednisone, with improvement in FLAIR signal abnormality and resolution of symptoms by six months (Figure 1, Panel B).

One year later, she experienced identical symptoms on the contralateral side of her face. MRI revealed FLAIR signal abnormality and enhancement of the left trigeminal root entry zone (Figure 1, Panel C). The differential diagnosis at this time included inflammatory lesion, neoplastic process (e.g., lymphoma), or viral etiology. During this period, a PET-CT was not performed. New viral studies at this time included Lyme IgG/IgM antibody testing and western blot, which were negative for Lyme disease. Her inflammatory workup was notable for antinuclear antibody positivity and elevated ribonucleoprotein antibody. A lumbar puncture was obtained while off steroids which showed modest pleocytosis: white blood cells 13 cells/µL, red blood cells 350 cells/µL, glucose 57 mg/dL, protein 67 mg/dL, negative cytology, and negative oligoclonal bands. She was started on a prednisone taper with minimal improvement in symptoms.

Four months later, the patient experienced right facial palsy. MRI revealed the resolution of the previous abnormality surrounding the left trigeminal root entry zone, now with FLAIR signal abnormality and abnormal enhancement within the right facial nerve (Figure 1, Panel D). A working diagnosis at this time was inflammatory pseudotumor of the trigeminal and facial nerves. Inflammatory and neoplastic processes such as sarcoid and lymphoma remained on the differential. She was started on another prednisone taper. The patient’s facial weakness resolved with the resolution of enhancement on the six-month MRI (Figure 1, Panel E).

**Figure 2:**
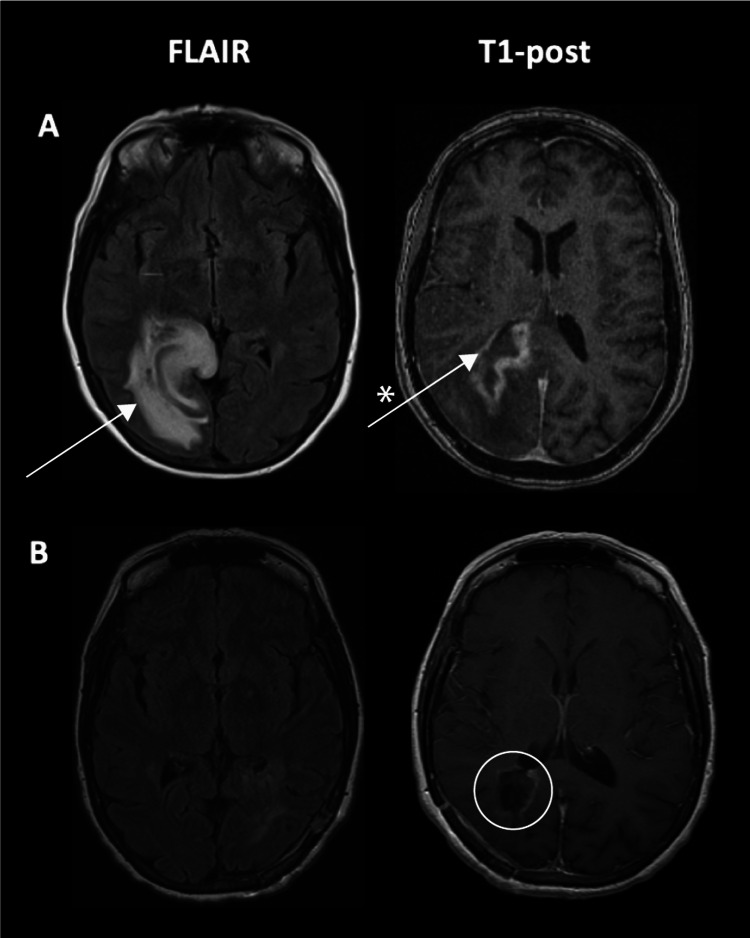
Right parietal mass: Preoperative and postoperative MRI. Two years after symptom onset, the patient developed left homonymous hemianopsia and was found to have a peripherally enhancing and partially hemorrhagic mass centered in the right parietal lobe (Arrow A), extending to the posterior corpus callosum and occipital and temporal lobes, with ependymal and subependymal involvement along the right lateral ventricle (Arrow * A). She was taken for a right parietal craniotomy. Her postoperative MRI showed small residual enhancement along the medial aspect of the resection cavity (Circle B) with near-complete resection of the tumor (B).

She developed visual disturbance one year later. Neurologic examination was notable for left homonymous hemianopia. Contrast-enhanced MRI identified a peripherally enhancing 4 x 2 cm, partially hemorrhagic mass involving the right parietal lobe and splenium of the corpus callosum, with vasogenic edema and cortical and subependymal involvement concerning for high-grade glioma (Figure 2, Panel A). The patient’s prior diagnosis of inflammatory pseudotumor and new brain mass were favored to be separate processes.

After informed consent was obtained, the patient was taken for a right parietal craniotomy. Intraoperatively, the brain was slightly swollen with a normal cortical appearance. The tumor was gray-pink and hypervascular. It had a heterogeneous texture with regions of necrosis and firm fibrinous portions.

Postoperative MRI revealed near-complete resection (Figure 2, Panel B). Fluorine 18 (18F)-fluorodeoxyglucose (FDG)-PET did not identify areas of uptake elsewhere in the body, consistent with disease confined to the CNS. She completed two four-dose courses of rituximab monotherapy and was started on maintenance dosing every 12 weeks. The serum EBV polymerase chain reaction remained negative. Subsequent post-treatment MRIs did not identify disease progression at three, six, 12, 24, and 36 months after surgery. Repeat 18F-FDG-PET at 36 months did not show abnormal areas of uptake.

Pathologic findings

**Figure 3:**
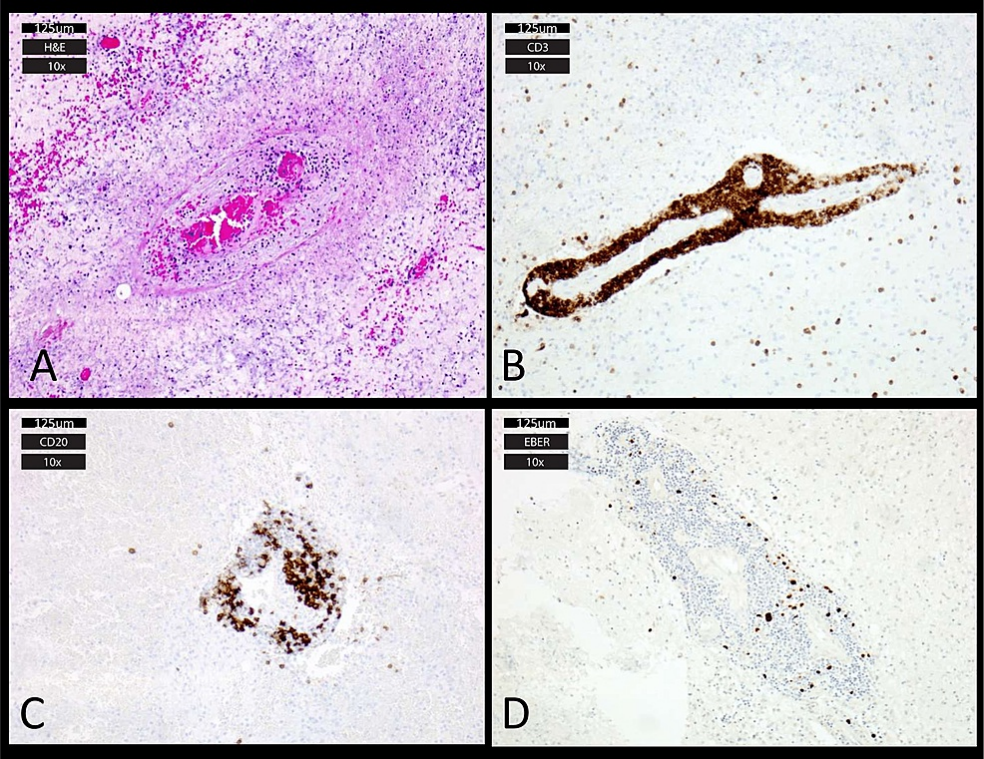
Histology and immunohistochemistry. Hematoxylin and eosin staining revealed perivascular lymphoid cuffs, vessel wall destruction, and thrombosis with associated necrosis (A). Immunohistochemistry showed CD3-positive T cells concentrated in a perivascular fashion (B). CD20-positive B cells were scattered and clustered (C). 5-20 EBER-positive cells were found per high-powered field, consistent with a diagnosis of grade 2 lymphomatoid granulomatosis (D).

On histology, there were perivascular lymphoid cuffs composed of numerous small irregular lymphocytes as well as areas of vessel wall destruction, thrombosis, and necrosis (Figure 3). Focal lymphocytic infiltration into the brain parenchyma was also seen. On immunohistochemistry, an inflammatory infiltrate composed of B cells and T cells in a perivascular distribution was present, with 5-20 EBV-positive cells per high-powered field (Figure 3). Overall, the histology was consistent with grade 2 lymphomatoid granulomatosis.

## Discussion

Isolated CNS lymphomatoid granulomatosis is a rare diagnosis with no standard of care. Interestingly, our patient first presented with trigeminal and facial nerve involvement separated in time, later presenting as a mass. Combined diffuse and mass-like presentations have been reported in 6% of cases [4].

Prior reports of isolated CNS lymphomatoid granulomatosis have reported symptomatic cranial nerve involvement without cranial nerve enhancement. Our patient had enhancement of the trigeminal and facial nerves which corresponded to the onset and regression of neurologic symptoms. This has been demonstrated in systemic lymphomatoid granulomatosis with CNS involvement but not in isolated CNS lymphomatoid granulomatosis [3,4].

Systemic lymphomatoid granulomatosis treatment paradigms and survival depend on staging [6]. Our patient initially responded to steroid monotherapy, which has previously been reported in the literature [7]. After resection of the right parietal mass, she underwent monotherapy with rituximab alone, an anti-CD20 monoclonal antibody. Case reports of lymphomatoid granulomatosis treatment using rituximab alone have shown survival of up to 36 months at the time of publication [8]. Another treatment paradigm considered was treating the patient’s isolated CNS lymphomatoid granulomatosis as a primary CNS lymphoma, in which the patient would receive methotrexate, rituximab, and temozolomide.

Historical data has shown that patients with systemic disease and CNS involvement have reduced survival [7]. When isolated to the CNS, survival has ranged from months up to 18 years and is limited by short follow-up and inconsistent reports of grading. The patient is currently doing well without progression of disease or new neurologic deficit more than three years after surgical intervention.

## Conclusions

Our case report highlights the challenges in lymphomatoid granulomatosis diagnostic workup and shows favorable survival for mass lesions after resection and rituximab monotherapy. Clinicians should be suspicious of lymphoproliferative disorder when an intracranial mass is preceded by steroid-responsive cranial neuropathies.
